# First-principles investigation of adsorption behaviors of small molecules on penta-graphene

**DOI:** 10.1186/s11671-018-2687-y

**Published:** 2018-09-03

**Authors:** Hongbo Qin, Chuang Feng, Xinghe Luan, Daoguo Yang

**Affiliations:** School of Mechanical and Electronic Engineering, Guilin University of Electronic Technology, No. 1, Jinji Road, Guilin, 541004 People’s Republic of China

**Keywords:** Penta-graphene, Gas molecule, Adsorption behavior, Gas sensing, First principles

## Abstract

The gas-adsorption behaviors of small molecules CO, H_2_O, H_2_S, NH_3_, SO_2_, and NO on pristine penta-graphene (PG) were investigated using first-principles calculations to explore their potential for use as advanced gas-sensing materials. Results show that, except for CO, H_2_O, H_2_S, NH_3_, and SO_2_ are physically adsorbed on the surface of penta-graphene with considerable adsorption energy and moderate charge transfer, while NO is prone to be chemically adsorbed on the surface of penta-graphene. Moreover, the electronic properties of PG can be effectively modified after H_2_O, H_2_S, NH_3_, SO_2_, and NO are adsorbed, and penta-graphene has potential for using in gas sensors via the charge-transfer mechanism.

**Electronic supplementary material:**

The online version of this article (10.1186/s11671-018-2687-y) contains supplementary material, which is available to authorized users.

## Background

Gas sensing, especially polluted or toxic gas sensing, has always been a focus of research associated with applications in the fields of environmental pollution monitoring, industrial control, agricultural production, and medical diagnosis [1]. Recently, increasingly more two-dimensional materials have been predicted and synthesized [2–4]. Two-dimensional materials have been extensively investigated and employed as gas-sensing elements since they exhibit fascinating properties, such as large surface area, ultrahigh carrier mobility, and low electrical noise [5]. It has been reported that the electronic properties of two-dimensional materials may be changed after some specific gas molecules are adsorbed on them [6, 7].

Among two-dimensional materials, particularly graphene and its analogues have attracted attention by virtue of their remarkable physical properties and potential for applications in both nanoelectronics and nanomechanics [8–12]. Although graphene has widely been regarded as one of the most suitable host materials for next-generation electronic devices [13], the stable *sp*^2^ hybridization of carbon bonds and zero-gap character render it inefficient for gas adsorption, which is disadvantageous for the design of gas sensors. Moreover, graphene is a conductor having excellent electrical conductivity [8]. Compared with semiconductors, it is difficult to measure the resistance information during the process of gas adsorption and graphene is insensitive to the concentration variations of gases. Thus, graphene needs to be functionalized for opening the band gap and acting as a semiconductor [10]. For the limitation of experimental characterization, the adsorption behaviors of molecules on the surface of graphene have been widely investigated by first-principles calculation, which are meaningful for the application of graphene [14–16].

Penta-graphene (PG), which can be exfoliated from bulk T12 carbon, is one of the most recently proposed graphene allotropes, and it consists of repeating carbon pentagon structures [17]. Some investigations have predicted that PG is stable with fixed lattice constants [17, 18]. It has a honeycomb structure and is a promising metal-free, low-cost catalyst for low-temperature CO oxidation [19]. Nitrogen-doped PG displays very high catalytic activity and is more competitive with many metal-based and carbon-based catalysts for low-temperature CO oxidation owing to the very small energy barrier of the rate-limiting step [20]. It has also been reported that transition-metal-doped PG is a potential hydrogen-storage material [21]. Furthermore, unlike graphene, PG is an intrinsic quasi-direct-band-gap semiconductor with a band gap in the range 1.52–4.48 eV [8, 17, 22], implying an enormous potential for application in semiconductor gas sensors. In addition, PG has a unique hybrid bond structure containing both *sp*^3^ and *sp*^2^ carbon bonds. Owing to the tetrahedral character of the *sp*^3^ hybridization of carbon bonds, PG is not ideally planar, but rather oscillates out-of-plane in a periodic corrugated manner [17], indicating more possible positions for gas adsorption as a sensing element.

Investigations of the interaction between small gas molecules and pristine PG have been scarce until now. Owing to the limitation of experimental methods, in this study, density functional theory (DFT) calculations were carried out to investigate the adsorption behavior of small gas molecules (i.e., CO, H_2_O, H_2_S, NH_3_, SO_2_, and NO) on the novel carbon material PG. This research will help workers analyze and predict the performance of PG applied in gas sensors.

## Methods

**Fig. 1:**
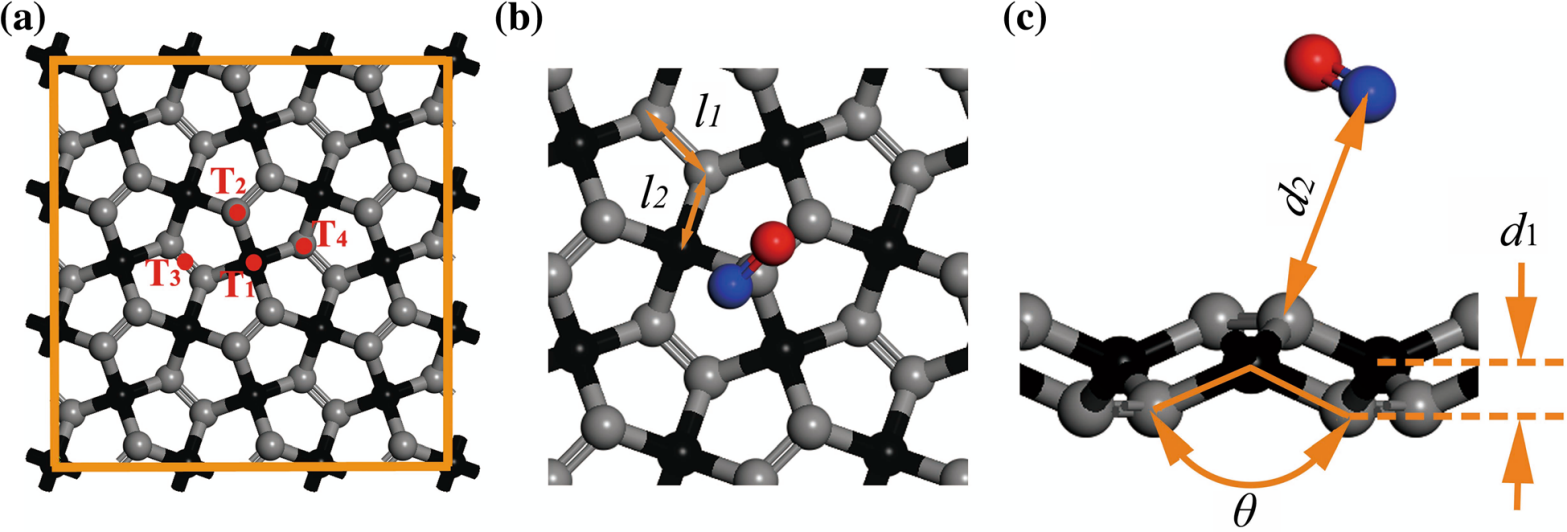
Structure and geometry of PG: **a** 3 × 3 supercell, **b** front view of unit cell, and **c** side view of the adsorbate-PG atoms. The distance between two C2 atoms, the distance between C1 and C2 atoms, the thickness of PG, and the C2-C1-C2 angel are defined as *l*_1_, *l*_2_, *d*_1_, and *θ*, respectively

In this study, the calculations of structural optimizations were carried out by first-principles calculations based on DFT [23] as implemented in Dmol^3^ code [24]. It is supposed that the local-density approximation (LDA) is propitious to study gas-molecule-adsorption systems [25, 26], and the LDA-PWC was selected for the structural optimizations in this investigation. To avoid neglecting the van der Waals interactions in the study gas-molecule adsorption, the method of Ortmann, Bechstedt, and Schmidt [27] was employed. The 2 × 2 × 1 Monkhorst-Pack mesh [28] was used for the Brillouin-zone integration, in which the self-consistent field tolerance was set as 1 × 10^− 5^ Ha. The system would reach the ground state when the convergence precision of energy for the maximum energy change, the maximum force, and the maximum displacement were 1 × 10^− 5^ Ha, 0.002 Ha/Å, and 0.005 Å, respectively. Multi-core parallel computing was carried out [29] and spin polarization was applied in the calculations of the adsorption of NO. A 3 × 3 supercell with a vacuum space of 30 Å [21] was modeled based on the unit cell containing two *sp*^3^-hybridized carbon (C1) atoms and four *sp*^2^-hybridized carbon (C2) atoms [17], see Fig. 1, where C1 and C2 atoms are distinguished as black and gray spheres, respectively. Gas molecules were located horizontal to the substrate at an initial distance of 3.5 Å. To obtain the most favorable adsorption positions of the gas molecules, four possible sites were investigated, namely the top of the C1 atom (T1), the top of the C2 atom (T2), the middle of the grooves in PG (T_3_), and the opposite position of T_2_ (T_4_), as shown in Fig. 1.

In order to quantitatively evaluate the adsorption capability of the system, in addition to LDA-PWC, adsorption energy (*E*_a_), charge transfer (*Q*), and adsorption distance (*d*_2_, as illustrated in Fig. 1) were calculated using the generalized gradient approximation (GGA) functionals with Perdew-Wang 1991 (PW91) and Perdew-Burke-Ernzerh (PBE) in the study. *d*_2_ is defined as the nearest atomic distance between the PG and the gas molecule in the equilibrium state. *Q* denotes the Mulliken charge of gas molecule [30, 31], and a negative value means charge transfer from PG to the gas molecule. The adsorption energy is calculated by

1 $$ {E}_a\kern0.5em =\kern0.5em {E}_{\left(\mathrm{PG}\kern0.5em +\kern0.5em \mathrm{molecule}\right)}\kern0.5em -\kern0.5em {E}_{\mathrm{PG}}\kern0.5em -\kern0.5em {E}_{\mathrm{molecule}}, $$

where *E*_(PG + molecule)_, *E*_PG_, and *E*_molecule_ are the total energies of the adsorbate-PG equilibrium system, isolated PG, and isolated gas molecule, respectively. In the computations of electronic structures of the adsorption systems, the DFT calculations using the PBE exchange-correlation functional with the GGA have been employed for higher accuracy [2, 32].

## Results and discussion

**Fig. 2:**
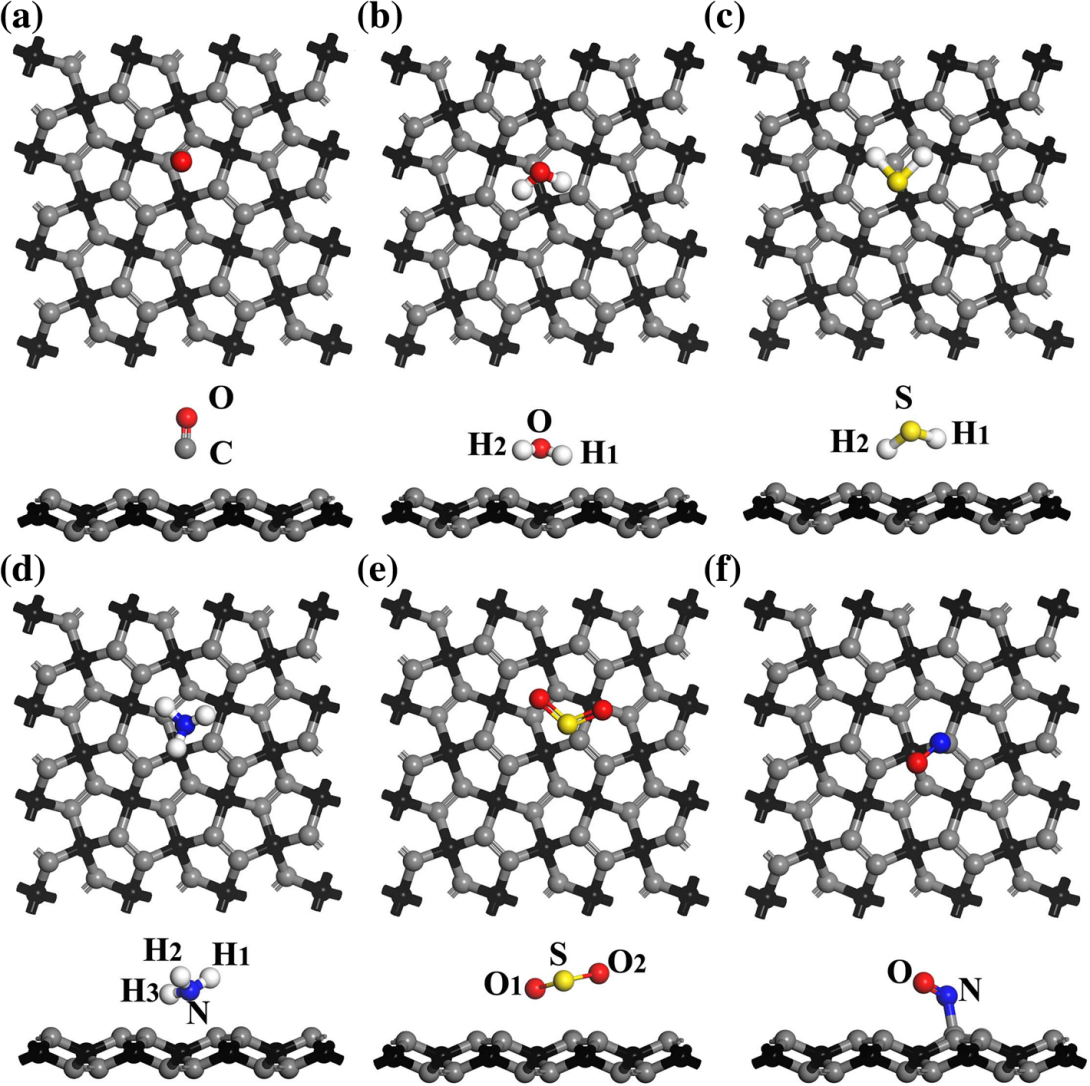
Top and side views of the most favorable adsorption configurations for **a** CO, **b** H_2_O, **c** H_2_S, **d** NH_3_, **e** SO_2_, and **f** NO on PG. The gray, red, white, yellow, and blue spheres represent C, O, H, S, and N atoms, respectively

After structural optimization, the calculated structural parameters of pristine PG reported in this paper (*l*_1_ = 1.342 Å, *l*_2_ = 1.551 Å, *θ* = 133.9°, *d*_1_ = 0.612 Å) were found to be consistent with those of previous work [17]. By selecting the lowest *E*_(PG + molecule)_ or *E*_a_ at the four adsorption positions T_1_ to T_4_ (see Table S1 of Additional file 1), the most favorable adsorption configurations for gases on monolayer PG are plotted in Fig. 2, and the most favorable adsorption positions (i.e., T_1_, T_2_, T_3_, or T_4_) are listed in Table 1. The calculated results in the following text are obtained based on these most favorable adsorption configurations. Recent studies have revealed that monolayer InSe, graphene, and blue phosphorus hold great promise for using in gas sensors [14, 33, 34]. In the present study, the calculated *E*_a_ values of CO, H_2_O, and NH_3_ are − 0.531, − 0.900, and − 1.069 eV (see Table 1), respectively, while they are − 0.120, − 0.173, and − 0.185 eV, respectively, for InSe [33]. For CO, H_2_O, NH_3_, and NO on the surface of graphene, the calculated *E*_a_ values are − 0.014, − 0.047, − 0.031, and − 0.029 eV, respectively [14]. Meanwhile, *E*_a_ values for H_2_S and SO_2_ gases on PG are − 1.345 and − 1.212 eV, respectively, which are much larger than − 0.14 and − 0.20 eV, respectively, for blue phosphorus [34]. Obviously, the calculated *E*_a_ values of these gas molecules on PG are much larger than these obtained from the other materials, indicating that these gas molecules are easy to be adsorbed on the surface of PG [35]. Considering that the calculated *E*_a_ value of CO is much smaller than that of other gases, the adsorption of CO may be the weakest. Meanwhile, the adsorption energy of NO in Table 1 is smaller than some of those physisorbed (non-covalent) adsorbates, such as H_2_S, NH_3_, and SO_2_. This can be explained by the reason that, different with physical adsorption, the chemical adsorption of NO induces obvious deformation of PG, which consumes extra energy and reduces the calculated adsorption energy *E*_a_, as introduced in Additional file 1. Similarly, obvious deformation can also be observed in the chemical adsorption of NO_2_ on the surface of antimonene [5], which may result in the relatively low adsorption energy. Furthermore, except for NO, the values of *d*_2_ listed in Table 1 are obviously larger than the sum of covalent radii of the corresponding atom in the gas molecule and the C atom in the PG (i.e., *l*_C-O_ = 1.38 Å, *l*_C-H_ = 1.07 Å, *l*_C-N_ = 1.46 Å, *l*_C-S_ = 1.78 Å) [36], revealing that these gas molecules tend to be physically adsorbed. Regarding NO, the value of *d*_2_ in the adsorption system is 1.541 Å, which is in the covalent-bonding range, indicating that chemical bonds may exist in this case.

**Table 1:** Calculated *E*_a_, *Q*, and *d*_*2*_ of small gas molecules adsorbed on PG

Gas molecule	Site	*E*_a_ (eV)	*Q* (e)	*d*_2_ (Å)
CO	T_1_	− 0.531	0.023	2.702 (C)
H_2_O	T_2_	− 0.900	0.082	2.026 (H_1_)
H_2_S	T_3_	− 1.345	0.133	1.930 (H_2_)
NH_3_	T_2_	− 1.069	0.169	2.315 (N)
SO_2_	T_2_	− 1.212	− 0.109	2.374 (S)
NO	T_1_	− 0.945	− 0.030	1.541 (N)

Previous studies on InSe and boron phosphide have shown that adsorbed molecules change the resistivity of the substrate by acting as charge acceptors or donors [33, 37]. The *Q* values for CO, H_2_O, H_2_S, NH_3_, SO_2_, and NO on the surface of PG are 0.023, 0.082, 0.133, 0.169, − 0.109, and − 0.03 e (see Table 1), respectively, indicating that gases of CO, H_2_O, H_2_S, and NH_3_ donate electrons to PG while SO_2_ and NO obtain electrons from PG. It is worth mentioning that the *Q* values of CO, H_2_O, NH_3_, and NO on the surface of InSe are 0.006, 0.014, − 0.025, and 0.018 e, respectively [33], and those for CO, H_2_O, NH_3_, and NO on the surface of graphene are 0.012, − 0.025, 0.027, and 0.018 e, respectively [14], indicating that the gain or loss of electrons of the gas molecules on PG is more obvious than that on InSe and graphene. Moreover, although chemical adsorption may occur between NO and PG, the charge transfer is only − 0.030 e. This can be explained by the fact that the Mulliken charge distributions of N and O atoms are quite different (see Table S2 in Additional file 1) before and after chemical adsorption. The gain electrons of the O atom offset the loss electrons of the N atom, resulting in the total charge transfer of NO not being large, while the interaction of electrons between NO and PG is still obvious. According to the sensing mechanism of charge transfer of small gas molecules on the surface of InSe and InN [33, 38], it is speculated that PG may have great potential for use in gas sensors based on the charge-transfer mechanism.

Furthermore, calculations of the gas adsorption on PG were also performed using two GGA functionals, PW91 and PBE. The calculated values of *E*_a_, *Q*, and *d* for gas molecules on PG are listed in Table 2. The *E*_a_ values calculated by PW91 and PBE are smaller than that calculated by the LDA, whereas both PW91 and PBE give a larger *d*_2_ compared to the LDA. Different from the LDA, the GGA usually has a tendency to underestimate the adsorption energy and overestimate the bond distance, which is consistent with the results of previous works [26, 31]. It is worth mentioning that the tendencies of the results of these three functionals are consistent. For example, the calculated values of *E*_a_ of CO are smallest, and the calculated values of *E*_a_ of H_2_S and SO_2_ are larger than those of other gas molecules. Moreover, the calculated values of *d*_2_ of NO for the LDA, PW91, and PBE functionals are 1.514, 1.592, and 1.591 Å, respectively, which are all in the covalent-bonding range [36].

**Table 2:** Results for gas molecules on PG calculated by PW91 and PBE functionals

Gas molecule	Site	GGA-PW91			GGA-PBE		
		*E*_a_ (eV)	*Q* (e)	*d* (Å)	*E*_a_ (eV)	*Q* (e)	*d* (Å)
CO	T_1_	− 0.243	0.016	3.051 (C)	− 0.223	0.020	2.957 (C)
H_2_O	T_2_	− 0.595	0.034	2.372 (H)	− 0.404	0.036	2.214 (H)
H_2_S	T_3_	− 1.044	0.077	2.51 (H)	− 1.230	0.077	2.488 (H)
NH_3_	T_2_	− 0.637	0.084	2.966 (N)	− 0.475	0.077	2.804 (N)
SO_2_	T_2_	− 0.920	− 0.065	2.832 (S)	− 1.225	− 0.080	2.675 (S)
NO	T_1_	− 0.443	− 0.031	1.592 (N)	− 0.454	− 0.026	1.591 (N)

**Fig. 3:**
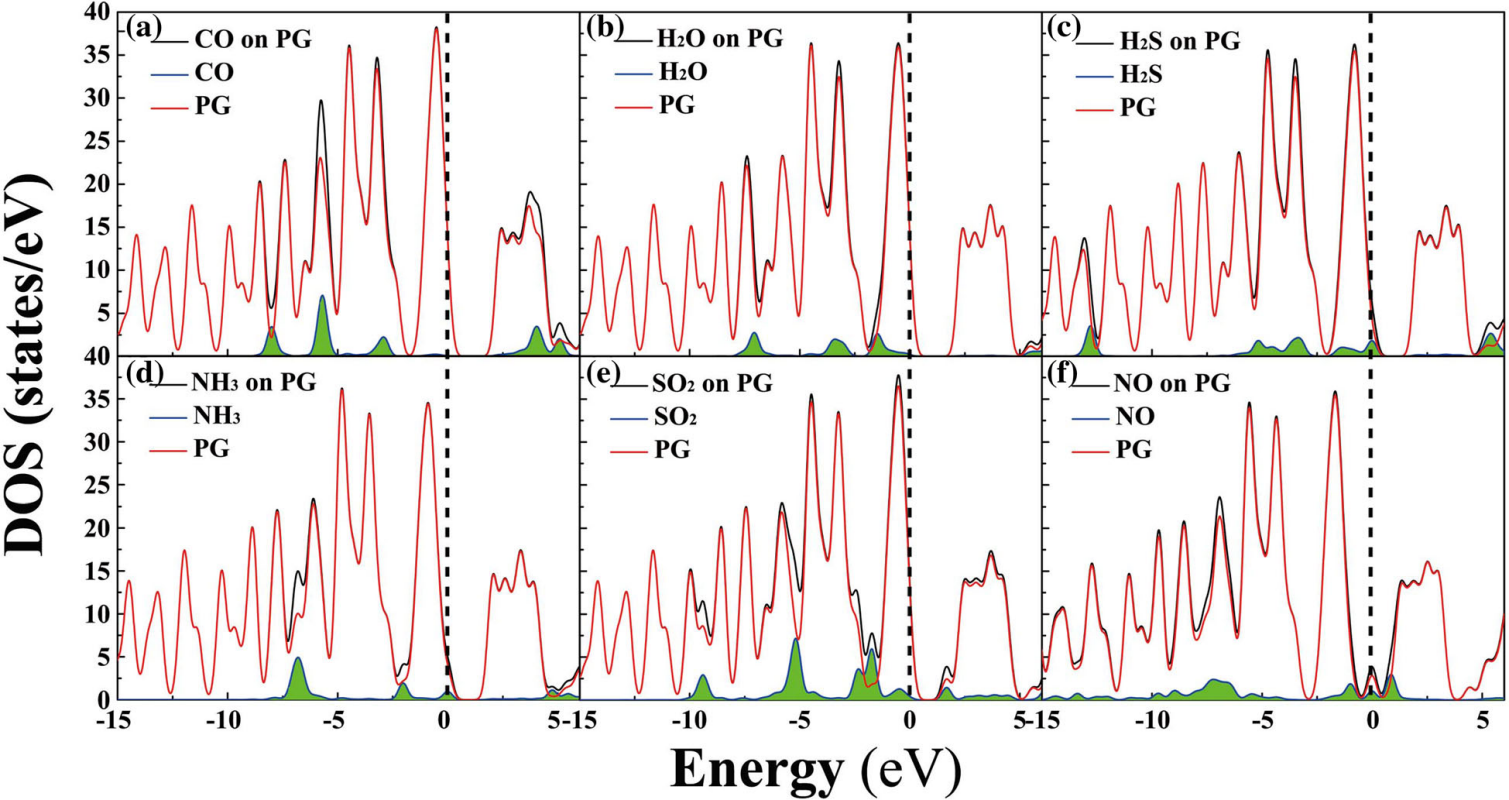
Total electronic density of states (DOS) for molecule-PG systems (black), and projected DOS for small molecules (blue line with green shadow) and PG (red) in the adsorption system: **a** CO, **b** H_2_O, **c** H_2_S, **d** NH_3_, **e** SO_2_, and **f** NO. The Fermi level is set to zero (see the dash line)

In order to obtain a better understanding on the influence of gas molecules on the electronic properties of PG, the density of state (DOS) of the molecule-PG system was calculated, see Fig. 3. Obviously, near the Fermi level (*E*_f_, e.g., in the range from − 2.5 to 2.5 eV), there are obvious contributions of the electronic levels of H_2_O, H_2_S, NH_3_, SO_2_, and NO to the adsorption systems, indicating that the existence of these gas molecules may have a great influence on the electronic properties of PG [5, 37]. For example, for H_2_S, an apparent contribution of electronic levels is located at 0 eV; see Fig. 3c. Regarding CO on the surface of PG, the orbital peaks of CO in the adsorption system are located at − 8.0, − 5.7, − 2.9, and 4.0 eV, and there is no obvious orbital contribution near *E*_f_. Additionally, band gap is also a critical factor in determining the electronic properties of materials [26, 34].

**Fig. 4:**
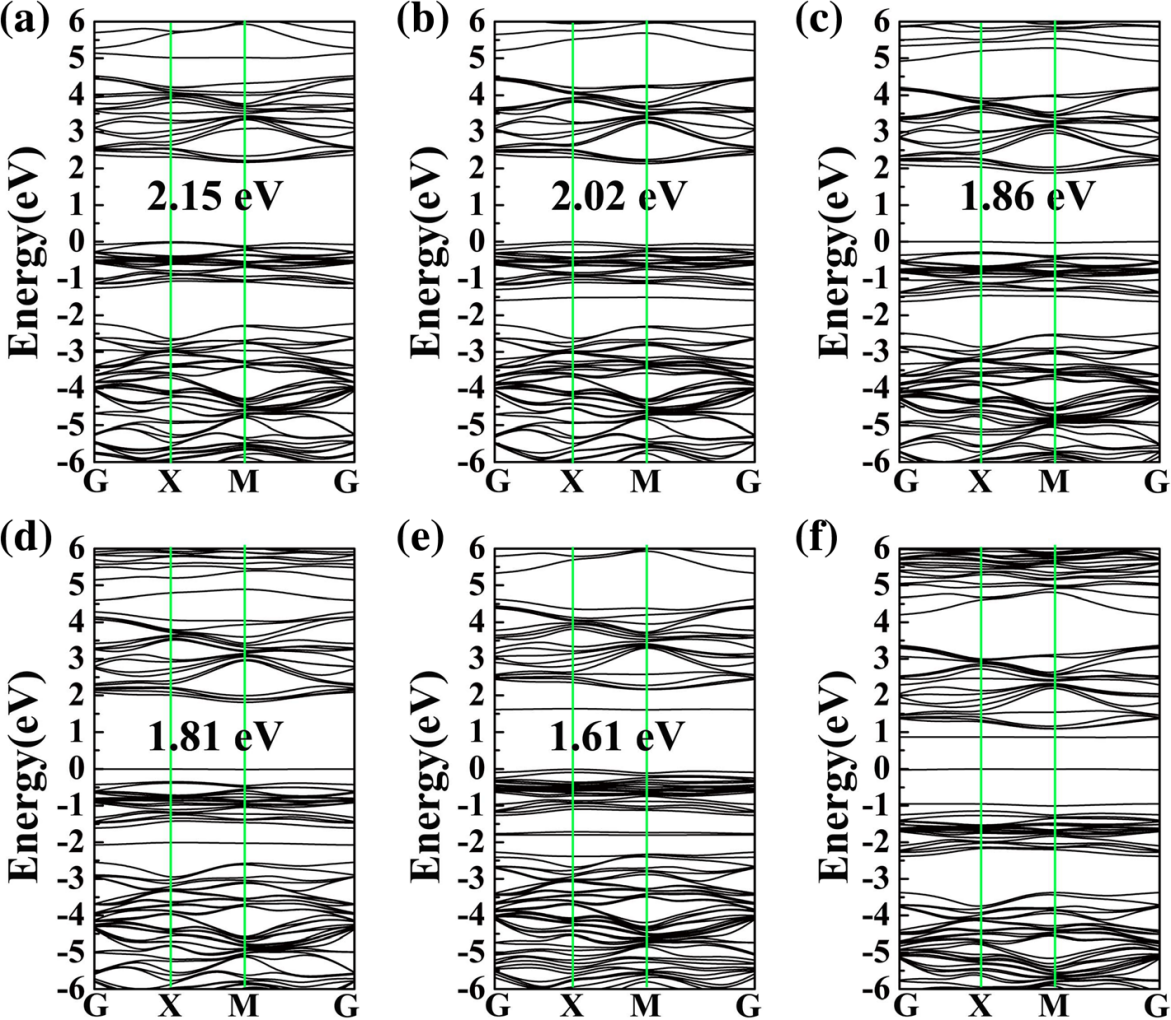
Band structures of CO (**a**), H_2_O (**b**), H_2_S (**c**), NH_3_ (**d**), SO_2_ (**e**), and NO (**f**) on the surface of PG

The band gaps and corresponding band structures of the adsorption systems are shown in Fig. 4, where the band gaps of CO, H_2_O, H_2_S, NH_3_, SO_2_, and NO on PG are 2.15, 2.02, 1.86, 1.81, 1.61, and 0 eV, respectively. As a contrast, the band gap of pristine PG is 2.21 eV (see Additional file 1: Figure S2). Clearly, except for CO, the gas adsorptions of H_2_O, H_2_S, NH_3_, SO_2_, and NO have obvious influence on the electronic properties of PG, and these are consistent with the results of DOS. All of these results may indicate that, except for CO, the electronic properties of PG can be effectively modified after H_2_O, H_2_S, NH_3_, SO_2_, and NO are adsorbed, which is critical for gas detection.

**Fig. 5:**
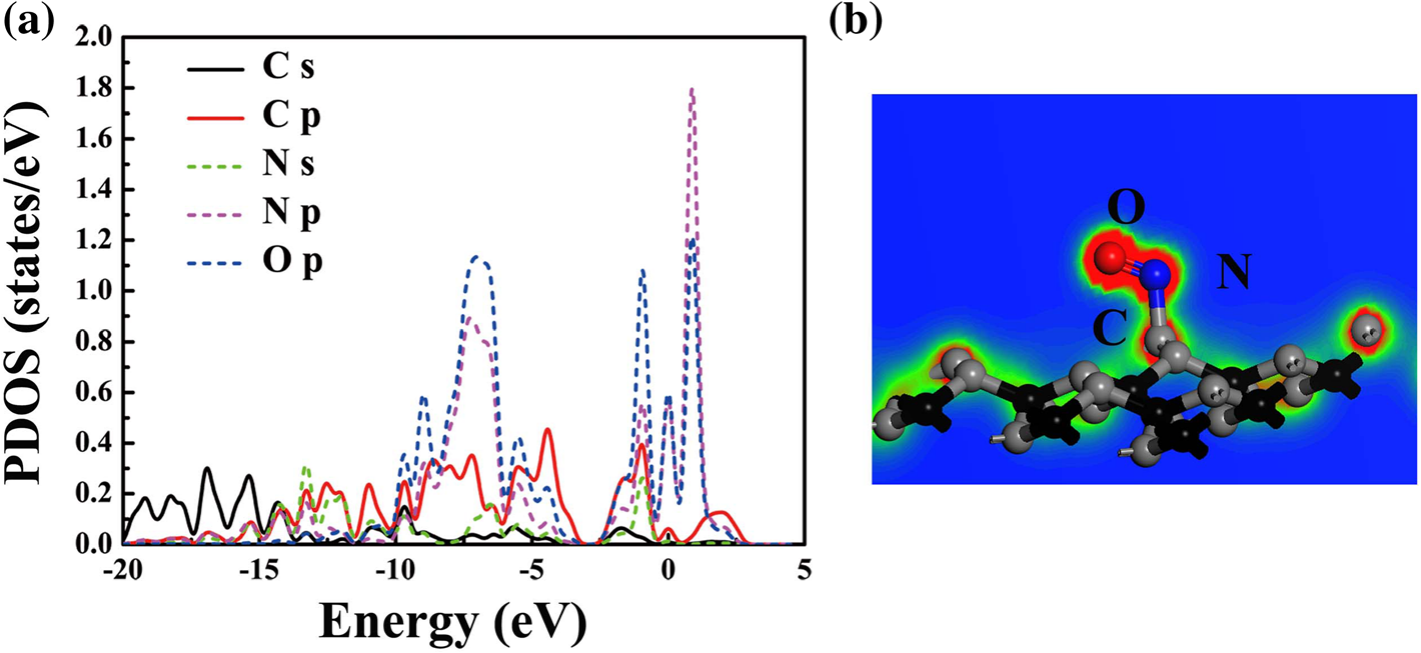
The atom projected DOS (**a**) and electron localization function (ELF) of NO-PG (**b**)

Considering that the *d*_2_ value of NO on PG is in the bonding range and that the electronic levels of NO in the system mainly localize around *E*_f_, it is speculated that a chemical adsorption occurs. Toward a deep understanding of the adsorption mechanism between NO and PG, the projected density of states (PDOS) of NO on PG and the electron localization function (ELF) are plotted in Fig. 5. Clearly, the peaks of the PDOS of N *p* and O *p* atoms are mainly located at − 6.9, − 0.9, 0, and 0.8 eV; thus there is an intra-molecule hybridization in NO, as shown in Fig. 5a. Meanwhile, orbital mixing between NO and the C atom of PG near the Fermi level can be observed, which is mainly contributed by the C *s*, C *p*, N *s*, N *p*, and O *p* orbitals. The orbital mixing induces a chemical bond between N in NO and C in PG, as displayed in Fig. 5b; thus, PG can be used for detecting or catalyzing NO gas [5, 39]. Further, in order to confirm the adsorption type of other gas molecules, ELFs of other adsorption systems are also calculated, see Additional file 1: Figure S3. Obviously, for CO, H_2_O, H_2_S, NH_3_ and SO_2_, there are no chemical bonds between the gas molecules and the substrate, indicating that the systems are trend to physical adsorption.

## Conclusions

In summary, H_2_O, H_2_S, NH_3_, and SO_2_ gases are physically adsorbed on monolayer PG with considerable adsorption energy and moderate charge transfer. For weak physical adsorption, small adsorption energy, and charge transfer, pristine PG is not suitable for detecting CO. For these gas molecules on the surface of PG, CO, H_2_O and H_2_S, and NH_3_ donate electrons to PG, while SO_2_ and NO obtain electrons from PG. Moreover, near the Fermi level, there are obvious contributions of the electronic levels of H_2_O, H_2_S, NH_3_, SO_2_, and NO to the DOS of the adsorption systems, indicating that the electronic properties of PG can be effectively modified after H_2_O, H_2_S, NH_3_, SO_2_, and NO are adsorbed. Furthermore, the adsorption of NO on PG shows a strong tendency of chemical adsorption, and thus PG can be used for detecting or catalyzing NO gas. Pristine PG, therefore, has great potential in gas-sensing applications.

## Additional file

Additional file 1: **Table S1.** The adsorption energies (*E*_a_) of small gas molecules of different initial positions adsorbed on PG. **Table S2.** The Mulliken charge distributions of the atoms of gas molecule before and after adsorption are defined as *C*_b_ and *C*_a_, respectively. **Figure S1.** The structures of SO_2_ (a) and NO (b) on PG, and the structure of PG after NO adsorption (c). **Figure S2.** The calculated band structure of the pristine PG using GGA-PBE method. **Figure S3.** The electron localization function (ELF) of (a) CO, (b) H_2_O, (c) H_2_S, (d) NH_3_, (e) SO_2_, and (f) NO on PG. (DOC 624 kb)

## Abbreviations

2D: Two-dimensional
DFT: Density functional theory
DOS: Density of states
ELF: Electron localization function
GGA: Generalized gradient approximation
LDA: Local-density approximation
PBE: Perdew–Burke–Ernzerh
PDOS: Projected density of states
PG: Penta-graphene
PW91: The Perdew-Wang 1991
PWC: Perdew–Wang correlational
