# Supplementary material for: First-principles investigation of adsorption behaviors of small molecules on penta-graphene
Source: Nanoscale Res Lett. 2018 Sep 3;13:264. doi: 10.1186/s11671-018-2687-y (PMC6120855; doi:10.1186/s11671-018-2687-y)
Supplement: Supplementary file 1 — Table S1. The adsorption energies (Ea) of small gas molecules of different initial positions adsorbed on PG. Table S2. The Mulliken charge distributions of the atoms of gas molecule before and after adsorption are defined as Cb and Ca, respectively. Figure S1. The structures of SO2 (a) and NO (b) on PG, and the structure of PG after NO adsorption (c). Figure S2. The calculated band structure of the pristine PG using GGA-PBE method. Figure S3. The electron localization function (ELF) of (a) CO, (b) H2O, (c) H2S, (d) NH3, (e) SO2, and (f) NO on PG. (DOC 624 kb) [file 11671_2018_2687_MOESM1_ESM.doc]

**Additional file 1**

For physical adsorption of gas molecule on PG, the structures of the substrates near the gas molecules have a slight deformation. For example, for SO_2_ on the surface of PG, in spite of its adsorption energy is as high as -1.212 eV, the two kinds of bond lengths (*l*_1_, *l*_2_, see Fig. S1 below) are 1.346 and 1.552 Å, respectively, which are close to the calculated structural parameters of pristine PG 1.342 and 1.551 Å, as shown in Fig. S1(a), indicating that the structure of PG changes slightly after the physic adsorption of SO_2_. However, for NO on the surface of PG, chemical bond can be formed, and the bond lengths of the substrate near NO have an obvious change (*l*_1_=1.439 Å, *l*_2_=1.597 Å, see Fig. S1(b) below). Considering the obvious change of the bond lengths of the substrate during chemical adsorption, it is speculated that the deformation of PG has influence on the calculated adsorption energy.

In order to get a further learn about the energy change after NO was adsorbed on PG, the energy of PG after the deformation was calculated. Results show that the energy of deformed PG is -55488.693 eV and the energy of pristine PG is -55489.106 eV, indicating that the deformation of PG absorbs an energy of 0.413 eV, which decreases the adsorption energy of NO calculated by Eq. (1) in the article.

**Table S1** The adsorption energies (*E*_a_) of small gas molecules of different initial positions adsorbed on PG.

| Gas molecule | T_1_ | T_2_ | T_3_ | T_4_ |
| --- | --- | --- | --- | --- |
| CO | -0.531 | -0.530 | -0.527 | -0.376 |
| H_2_O | -0.899 | -0.900 | -0.879 | -0.900 |
| H_2_S | -1.342 | -1.344 | -1.345 | -1.345 |
| NH_3_ | -1.063 | -1.069 | -1.046 | -1.045 |
| SO_2_ | -1.079 | -1.212 | -1.133 | -1.076 |
| NO | -0.945 | -0.894 | -0.893 | -0.665 |

**Table S2** The Mulliken charge distributions of the atoms of gas molecule before and after adsorption are defined as *C*_b_ and *C*_a_, respectively. The Mulliken charge of gas molecules before and after adsorption are defined as *Q*_b_ and *Q*_a_, respectively.

| Gas molecule | *C*_b_ | *C*_a_ | *Q_b_* | *Q_a_* |
| --- | --- | --- | --- | --- |
| CO | C:0.076  O:-0.076 | C:0.088  O:-0.065 | 0 | 0.023 |
| H_2_O | H_1_:0.272  O:-0.544  H_2_:0.272 | H_1_:0.285  O:-0.491  H_2_:0.288 | 0 | 0.082 |
| H_2_S | H_1_:0.198  S:-0.395  H_2_:0.198 | H_1_:0.227  S:-0.353  H_2_:0.259 | 0 | 0.133 |
| NH_3_ | N:-0.544  H_1_:0.181  H_2_:0.182  H_3_:0.181 | N:-0.515  H_1_:0.226  H_2_:0.226  H_3_:0.232 | 0 | 0.169 |
| SO_2_ | S:0.380  O_1_:-0.190  O_2_:-0.190 | S:0.390  O_1_:-0.249  O_2_:-0.250 | 0 | -0.109 |
| NO | N:0.018  O:-0.018 | N:0.095  O:-0.125 | 0 | -0.03 |


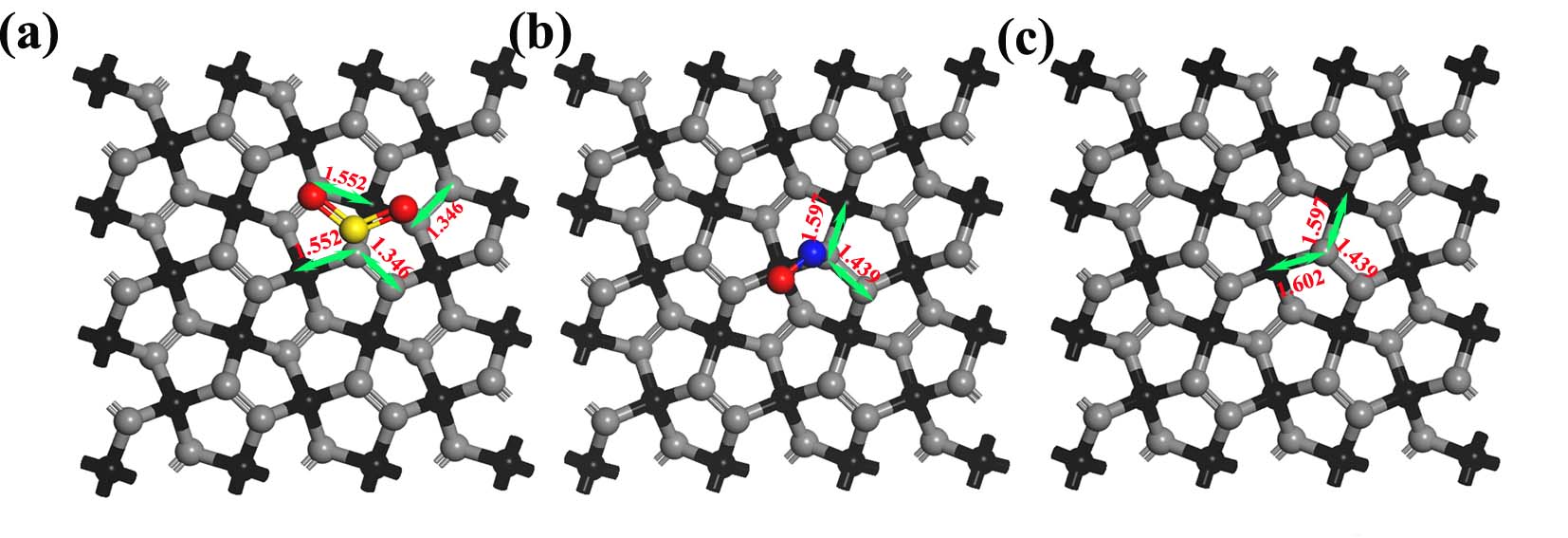


**Figure S1** The structures of SO_2_ (a) and NO (b) on PG, and the structure of PG after NO adsorption (c). The corresponding values of *l*_1_ and *l*_2_ are also plotted in red.


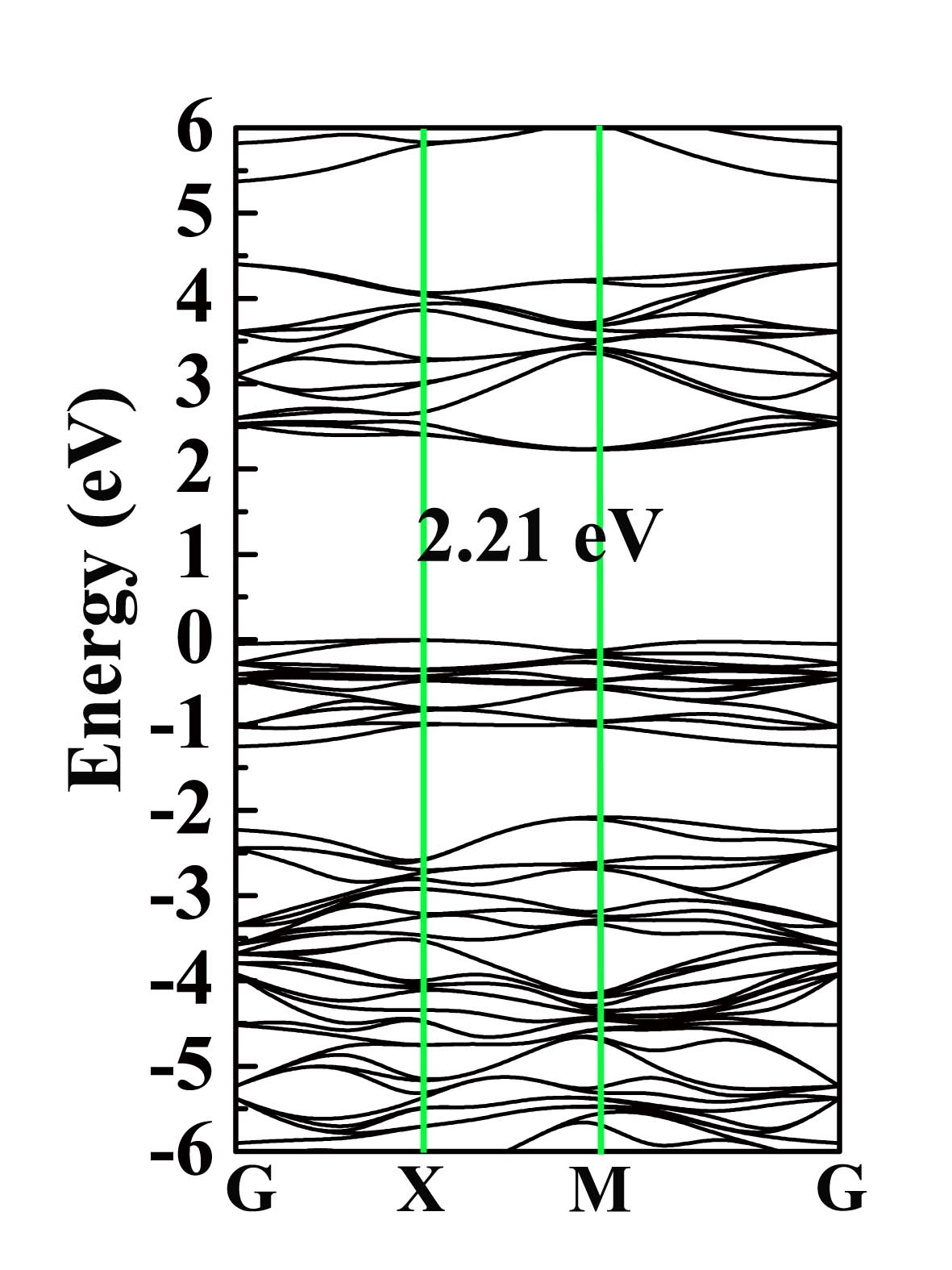


**Figure S2** The calculated band structure of the pristine PG using GGA-PBE method.


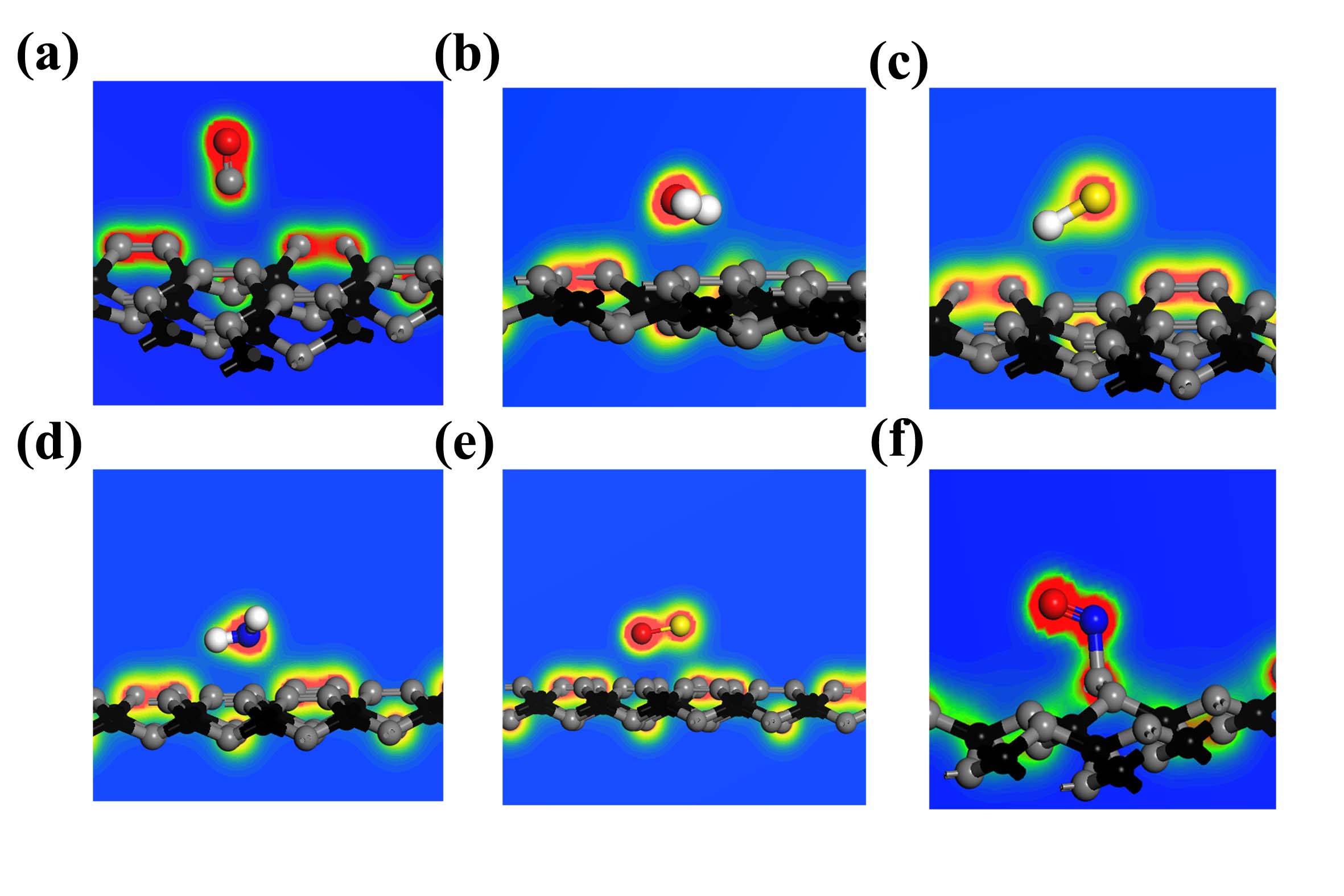


**Figure S3** The electron localization function (ELF) of (a) CO, (b) H_2_O, (c) H_2_S, (d) NH_3_, (e) SO_2_, and (f) NO on PG.
